# Dissecting Organ-Specific Aroma-Active Volatile Profiles in Two Endemic *Phoebe* Species by Integrated GC-MS Metabolomics

**DOI:** 10.3390/metabo15080526

**Published:** 2025-08-03

**Authors:** Ming Xu, Yu Chen, Guoming Wang

**Affiliations:** 1 Shanghai Lingang Fengxian Economic Development Co., Ltd., Shanghai 201413, China; xum@shlingang.com; 2 Jiangsu Key Laboratory for Conservation and Utilization of Plant Resources, Institute of Botany, Jiangsu Province and Chinese Academy of Sciences (Nanjing Botanical Garden Mem. Sun Yat-Sen), Nanjing 210014, China

**Keywords:** *Phoebe zhennan*, *Phoebe chekiangensis*, volatile organic compounds, rOAV, tissue-related aroma, flavoromics

## Abstract

**Background**: *Phoebe zhennan* and *Phoebe chekiangensis* are valuable evergreen trees recognized for their unique aromas and ecological significance, yet the organ-related distribution and functional implications of aroma-active volatiles remain insufficiently characterized. **Methods**: In this study, we applied an integrated GC-MS-based volatile metabolomics approach combined with a relative odor activity value (rOAV) analysis to comprehensively profile and compare the volatile metabolite landscape in the seeds and leaves of both species. **Results**: In total, 1666 volatile compounds were putatively identified, of which 540 were inferred as key aroma-active contributors based on the rOAV analysis. A multivariate statistical analysis revealed clear tissue-related separation: the seeds were enriched in sweet, floral, and fruity volatiles, whereas the leaves contained higher levels of green leaf volatiles and terpenoids associated with ecological defense. KEGG pathway enrichment indicated that terpenoid backbone and phenylpropanoid biosynthesis pathways played major roles in shaping these divergent profiles. A Venn diagram analysis further uncovered core and unique volatiles underlying species and tissue specificity. **Conclusions**: These insights provide an integrated reference for understanding tissue-divergent volatile profiles in *Phoebe* species and offer a basis for fragrance-oriented selection, ecological trait evaluation, and the sustainable utilization of organ-related metabolic characteristics in breeding and conservation programs.

## 1. Introduction

Volatile organic compounds (VOCs) play crucial roles in plant physiology and ecology, acting as key mediators in plant–environment interactions, defense responses, and inter-organism communication [1,2]. Beyond their ecological functions, VOCs contribute significantly to the flavor and aroma quality of foods, spices, herbal medicines, and aromatic wood products [3]. The composition and abundance of VOCs are highly species- and organ-related and are influenced by genetic background, tissue type, developmental stage, and environmental factors [4,5]. Recent advances in plant metabolomics have enabled the large-scale profiling of volatile metabolites, providing new insights into the biochemical diversity and evolutionary significance of plant aroma profiles [6]. Understanding the patterns and mechanisms of VOC accumulation across species and organs is essential for exploring biodiversity, guiding aroma-oriented breeding, and optimizing the use of aromatic and medicinal plants.

The genus *Phoebe* (Lauraceae) includes over 200 species worldwide, many of which are valued for their ecological roles, fragrant wood, and horticultural appeal [7]. Among them, *P. zhennan* and *P. chekiangensis* are two important endemic species in China. *P. zhennan*, mainly distributed in Sichuan, Chongqing, and Guizhou Provinces, is historically renowned for its durable, aromatic timber used in ancient palatial architecture [8,9]. *P. chekiangensis*, native to Zhejiang and neighboring regions, is valued for its dense canopy, ornamental characteristics, and fragrant wood and is listed as a nationally protected species due to habitat loss and overuse [10,11]. Both species are widely planted in urban landscapes and ecological restoration because of their evergreen foliage, shade tolerance, and pest resistance. Their wood and foliage emit distinct pleasant aromas, mainly derived from volatile secondary metabolites such as monoterpenes, sesquiterpenes, and phenylpropanoids [12,13]. While previous studies have explored terpenoid-rich extracts from *Phoebe* wood for antimicrobial and antioxidant activities, detailed volatile metabolite profiles of *P. zhennan* and *P. chekiangensis*, especially in non-wood tissues like seeds and leaves, remain scarce. To date, no study has systematically compared their aroma-active metabolomes across different organs, despite known organ- and species-related scent differences. A systematic metabolomic investigation is thus needed to clarify the biochemical basis of their aroma divergence and guide efforts to utilize their aroma and improve their germplasm.

Recent analytical advances have enabled the systematic profiling of plant volatiles [14]. Gas chromatography–mass spectrometry (GC-MS) remains a widely used and robust method for VOC analysis due to its high sensitivity and compound identification accuracy [15,16]. In addition, the relative odor activity value (rOAV) provides a quantitative approach to evaluate each compound’s sensory contribution by combining chemical concentration with odor thresholds [17,18]. rOAV-based screening has become increasingly important for identifying key aroma-active compounds in complex plant samples [19]. Moreover, aroma flavoromics is a strategy that combines metabolite annotation with known odor descriptors. It has gained traction in the study of aroma characteristics in foods, medicinal plants, and aromatic trees [20,21,22]. By integrating metabolomic data, aroma information, and pathway enrichment analyses such as Kyoto Encyclopedia of Genes and Genomes (KEGG) annotation, researchers can unravel species- and organ-related aroma traits with a high resolution.

However, despite the widespread application of metabolomics in herbaceous plants and fruits [15,16], its use in long-lived woody species like *Phoebe* remains relatively limited. Current studies mainly focus on wood extracts or essential oils, while the volatile metabolomes of seeds and leaves, which are organs with distinct ecological functions, remain largely uncharacterized. Given that wood volatiles in *Phoebe* species have been extensively characterized in prior studies, we focused this investigation on seeds and leaves to better understand the less-studied tissue-related aroma-active metabolites and their ecological implications. Clarifying these tissue-related volatile patterns is critical for linking metabolic signatures to organ functions, ecological interactions, and potential responses to abiotic stress. This represents a key knowledge gap limiting our understanding of tissue-related biosynthesis and the functional allocation of volatiles in Lauraceae species. To fill this gap, the present study aimed to (i) comprehensively profile volatile metabolites in the seeds and leaves of *P. zhennan* and *P. chekiangensis* using GC-MS-based untargeted metabolomics; (ii) identify key aroma-active compounds via rOAV screening; (iii) annotate compound-related sensory features through flavoromics analysis; and (iv) interpret the metabolic basis of species- and tissue-related aroma patterns using KEGG pathway enrichment and Venn diagram comparisons. These objectives together provide a new perspective on *Phoebe* species’ volatile diversity and the ecological implications of this in their application.

In trees, VOCs not only define species-related aromas but also support long-term environmental adaptation and biotic interactions [1,2]. Lauraceae members in particular show diverse volatile profiles due to their complex secondary metabolism. Despite their ecological and economic value, systematic interspecies and inter-organ comparisons are rare, hindering our understanding of their chemical ecology. In this study, we conducted an integrated GC-MS-based volatile metabolomic analysis of seeds and leaves from *P. zhennan* and *P. chekiangensis*, combining rOAV screening, KEGG pathway enrichment, and aroma descriptor annotation. This work provides a detailed view of tissue- and species-related aroma-active compounds and lays a basis for quality evaluation and future fragrance-oriented applications in *Phoebe* species.

## 2. Materials and Methods

### 2.1. Plant Materials

Mature seeds and leaves of two *Phoebe* species, *P. zhennan* (ZN) and *P. chekiangensis* (ZJN), were collected from healthy adult trees (approximately 5–6 years old) grown in the same experimental plantation at the Institute of Botany, Jiangsu Province and Chinese Academy of Sciences (Nanjing, China; 32°03′ N, 118°57′ E). A map showing the study area and the sampling points is provided in Figure S1. The plantation site lies within a northern subtropical monsoon climate zone, characterized by four distinct seasons, an average annual temperature of ~15.5 °C, and yearly precipitation between 1000 and 1200 mm. The trees were grown under uniform management conditions on loamy soil, and the elevation of the site is approximately 50 m above sea level. Leaf samples were collected from the upper sun-exposed branches of each tree at the peak of their functional activity, while seeds were harvested at full maturity based on external morphology and color. Seeds (designated as ZN1 and ZJN1) and leaves (designated as ZN2 and ZJN2) were collected from at least three individual trees to ensure biological representation during the peak period of seed maturity and leaf functionality. In total, ten samples were analyzed, comprising seed samples (ZJN1-1/2/3 and ZN1-1/2/3) and leaf samples (ZJN2-1/2 and ZN2-1/2). Each biological replicate was collected from a different individual tree to ensure independence. Due to limited material availability during sample preparation, only two replicates were included for leaf samples in each species, while three replicates were included for seeds.

### 2.2. Volatile Metabolite Extraction and GC-MS Analysis

Frozen samples (~0.2 g) were ground in liquid nitrogen and transferred into 20 mL headspace vials. Each vial was supplemented with 0.2 g NaCl and 20 μL of internal standard solution (10 μg/mL 2-octanol-d17). Volatile compounds were extracted using headspace solid-phase microextraction (HS-SPME) with a 120 μm DVB/CWR/PDMS SPME Arrow fiber at 60 °C for 15 min, followed by thermal desorption at 250 °C for 5 min. GC-MS analysis was performed using a DB-5MS capillary column (30 m × 0.25 mm × 0.25 μm, Agilent) with helium as the carrier gas (1.2 mL/min). The oven temperature was programmed from 40 °C (holding for 3.5 min) to 280 °C with ramping steps of 10, 7, and 25 °C/min. The mass spectrometer operated in EI mode (70 eV). The MS transfer line temperature was set at 280 °C, the ion source temperature at 230 °C, and the quadrupole temperature at 150 °C. The ionization voltage was 70 eV, scan speed was 2.5 scans/s, and the solvent delay was set to 5.0 min. Data were acquired in selected ion monitoring (SIM) mode. Metabolites were identified based on retention time and characteristic ions using an in-house reference library. Only compounds with a spectral similarity (cosine match) ≥80% and a retention index (RI) deviation ≤30 units were considered reliably annotated. Quantification was performed using the peak area of selected quantifier ions under selected ion monitoring (SIM) mode, with internal standard correction using 2-octanol-d17. The workflow followed the semi-quantitative protocol described by Yuan et al. (2022), in which selected ions were extracted under SIM mode and normalized using an internal standard for each sample [23]. Extraction, detection, and primary data processing were performed by Metware Biotechnology Co., Ltd. (Wuhan, China). To address potential issues arising from co-eluting isomers, metabolite identification was further supported by manual verification of diagnostic fragment ions and retention behavior. In cases in which stereoisomers could not be confidently resolved due to overlapping ion fragments or retention times, compounds were annotated as combined isomeric forms (e.g., “β-ocimene isomer”) following conservative reporting standards.

### 2.3. Data Preprocessing and Quantification

Raw GC-MS data were processed using MassHunter Workstation software (v. B.08.00, Agilent Technologies, Santa Clara, CA, USA) for peak integration and compound identification. Metabolite quantification was performed using a semi-quantitative internal standard method. For solid samples, relative concentrations (μg/g) were calculated based on the ratio of analyte to internal standard peak areas, adjusted by the amount and concentration of the internal standard. Missing values were imputed with one-fifth of the minimum value for each metabolite. Metabolites with a coefficient of variation (CV) < 0.5 across quality control (QC) samples were retained for downstream analysis. QC samples were prepared by pooling equal aliquots of all biological samples and injected periodically throughout the analytical run to monitor instrument stability. Relative odor activity values (rOAVs) were calculated using the equation: rOAVᵢ = *Cᵢ/Tᵢ*, where *Cᵢ* is the relative concentration of metabolite *i*, and *Tᵢ* is its odor threshold obtained from public databases or the literature [17,18]. Metabolites with rOAV ≥ 1 were considered aroma-active.

### 2.4. Functional Annotation and Biological Interpretation

All identified volatile metabolites were annotated using the KEGG database to assign metabolic pathways and functional categories. KEGG compound IDs were used to match metabolites to known biosynthetic and metabolic pathways, including terpenoid, fatty acid, alcohol, and aldehyde metabolism. Only metabolites with valid KEGG compound IDs were included in enrichment analysis. Some sesquiterpenes and norisoprenoids could not be mapped due to database limitations and were therefore excluded. KEGG enrichment analysis of differential metabolites was performed using a hypergeometric test, and significantly enriched pathways were visualized using bar plots or bubble charts. To interpret the potential sensory impact of differential volatile compounds, odor descriptors were assigned based on multiple public aroma databases, including The Good Scents Company (http://www.thegoodscentscompany.com (accessed on 1 November 2024)), Perflavory (http://perflavory.com (accessed on 1 November 2024)), Odour.org.uk (http://www.odour.org.uk/odour/index.html (accessed on 1 November 2024)), and Food Flavor Lab (http://foodflavorlab.cn/#/home (accessed on 1 November 2024)), as well as supporting information from peer-reviewed literature. These descriptors were used for qualitative reference to group aroma-related metabolites into categories, such as fruity, floral, green, sweet, herbal, and spicy aromas. Annotated aroma attributes were visualized using Sankey diagrams to highlight compound–odor relationships and to distinguish species-and organ-related sensory profiles.

### 2.5. Statistical Analysis and Data Visualization

All statistical analyses and visualizations were performed using R software (version 4.2.2) and GraphPad Prism 9. Prior to multivariate analysis, relative concentrations of volatile metabolites were log10-transformed and auto-scaled. Principal component analysis (PCA) was conducted using the ‘FactoMineR’ package to explore sample clustering patterns based on metabolite profiles. Hierarchical clustering analysis (HCA) was performed using the ‘pheatmap’ package with Euclidean distance and complete linkage to visualize differential metabolite accumulation among samples. Venn diagrams were constructed using the ‘VennDiagram’ package to identify shared and unique volatile metabolites between species and organs. Differentially accumulated volatile metabolites (DAMs) were screened using one-way ANOVA with false discovery rate (FDR) correction, and metabolites with adjusted *p* < 0.05 were considered significant. KEGG enrichment results were visualized using ‘ggplot2’, and Sankey diagrams of aroma descriptors were generated using RAWGraphs 2.0.

## 3. Results

### 3.1. QC Assessment and Global Metabolite Overview

**Figure 1:**
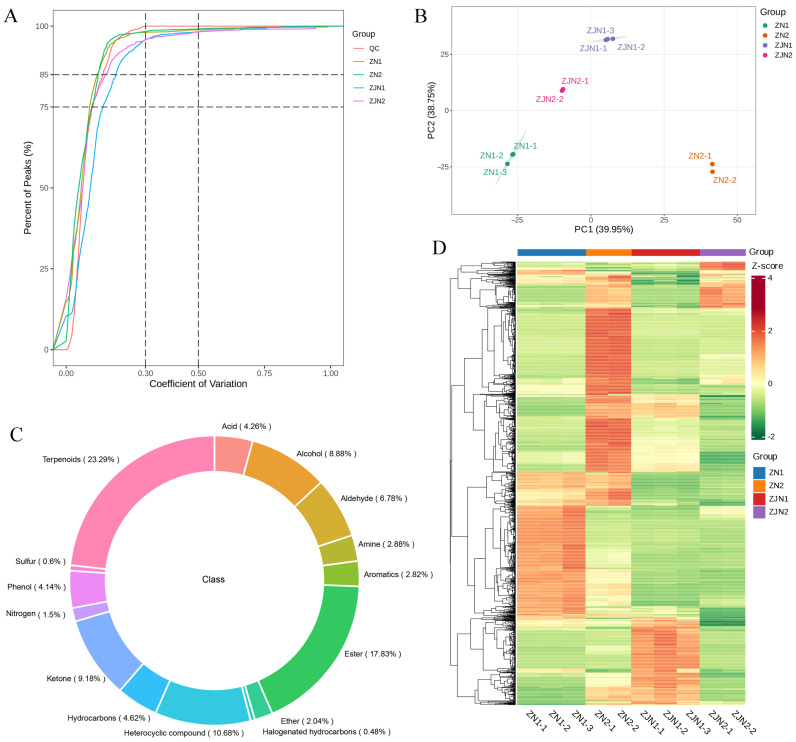
Overview of quality control and global metabolite profiling in *Phoebe* species. (**A**) Empirical cumulative distribution function (ECDF) showing the coefficient of variation (CV) of all detected peaks across sample groups. (**B**) PCA score plot based on mass spectrometry data from all samples and QC samples. The four sample groups include seeds and leaves of *P. zhennan* (ZN1, ZN2) and *P. chekiangensis* (ZJN1, ZJN2). (**C**) Circular composition plot showing the classification of the 1666 detected metabolites into different chemical categories. (**D**) Hierarchical clustering heatmaps of all detected metabolites and samples. Samples were arranged by biological group for clearer visualization, and only metabolites were hierarchically clustered.

To ensure data quality and reproducibility, we first conducted quality control analyses across all samples. The empirical cumulative distribution function (ECDF) of the coefficient of variation (CV) values showed that over 85% of the detected peaks had CVs below 0.5 across all groups (Figure 1A), demonstrating the high technical consistency of the GC-MS measurements and sample preparation. A PCA was performed based on the mass spectrometry profiles of all samples and QC samples. In the PCA score plot (Figure 1B), the QC samples clustered tightly together, further demonstrating the technical reproducibility. Clear separations among different groups were observed along PC1 (39.95%) and PC2 (38.75), suggesting distinct volatile metabolite profiles among the different tissues and species. A total of 1666 volatile metabolites were detected and classified into different chemical categories (Supplementary Table S1). The circular composition plot (Figure 1C) revealed that terpenoids, alcohols, aldehydes, ketones, and aromatic compounds were the predominant classes. An HCA was subsequently performed to visualize the overall differences in metabolite accumulation patterns (Figure 1D). Using all_heatmap_row_cluster, clustering was performed only on metabolites, highlighting the metabolites with similar abundance patterns across samples. These results confirmed the high quality and consistency of the data and provided a robust basis for subsequent differential metabolite analysis.

### 3.2. Identification of Key Aroma-Active Compounds Using rOAV Analysis

**Figure 2:**
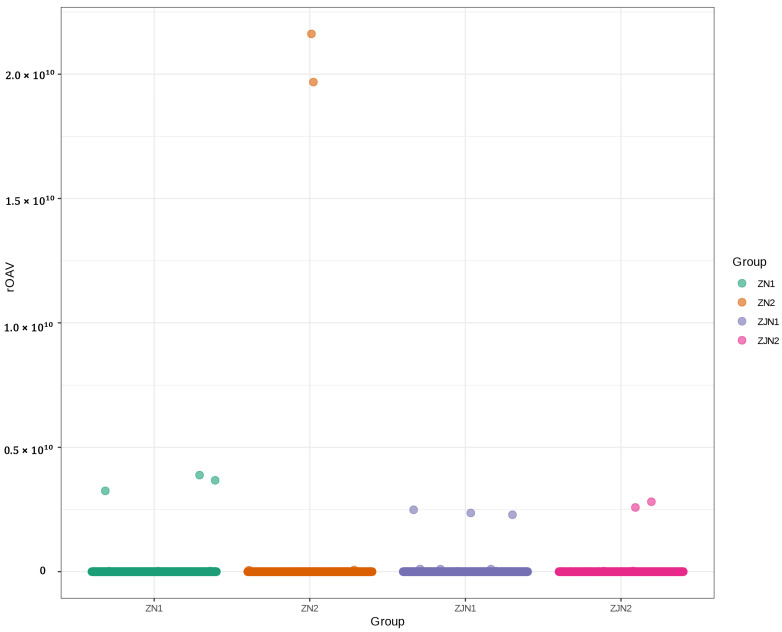
Scatter plot of relative odor activity values (rOAVs) of volatile metabolites across different groups. The x-axis represents different sample groups, and the y-axis indicates the rOAV values. Each point represents a metabolite, and colors denote different groups (ZN1, ZN2, ZJN1, ZJN2).

To identify the volatile metabolites contributing most significantly to the aroma profiles of different *Phoebe* samples, the relative odor activity value (rOAV) was calculated for each metabolite. The rOAV was determined as the ratio of the metabolite’s concentration to its odor threshold according to the published literature [17,18]. As shown in Figure 2, the majority of volatile metabolites exhibited relatively low rOAV values across all groups, whereas a few metabolites displayed exceptionally high rOAVs, particularly in ZN2. This indicates that these compounds may play dominant roles in defining the characteristic aroma profiles of the leaves. Notably, two metabolites (3-Cyclohexene-1-methanethiol,α,α,4-trimethyl- and β-damascone) in ZN2 exhibited rOAVs exceeding 2.0 × 10^10^, suggesting an extremely strong contribution to the overall aroma. Based on the threshold of the mean rOAV > 1, a total of 540 volatile metabolites were identified as key aroma-active compounds (Supplementary Table S2). These metabolites were considered to have substantial impacts on the overall sensory characteristics of the *Phoebe* samples and will be further analyzed to elucidate their biological and ecological significance.

### 3.3. Screening and Clustering Analysis of Differential Volatile Metabolites

**Figure 3:**
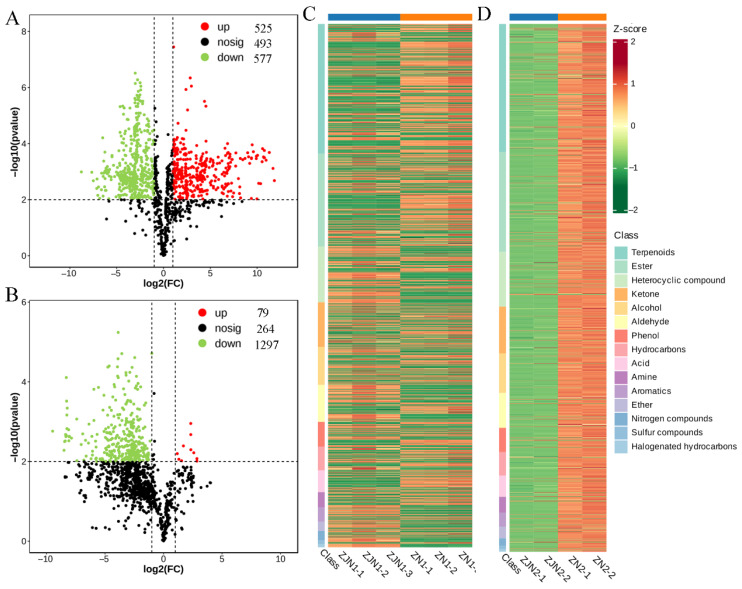
Differential volatile metabolite analysis between ZJN and ZN. (**A**) Volcano plot showing the differential volatile metabolites in seeds between ZJN1 and ZN1. (**B**) Volcano plot showing the differential volatile metabolites in leaves between ZJN2 and ZN2. In the volcano plots, red dots represent upregulated metabolites, green dots represent downregulated metabolites, and black dots represent non-significant metabolites. (**C**) Hierarchical clustering heatmap of significantly differential volatile metabolites between ZJN1 and ZN1. (**D**) Hierarchical clustering heatmap of significantly differential volatile metabolites between ZJN2 and ZN2. Heatmap categorized by primary metabolite classes.

To explore the volatile metabolite differences between *P. chekiangensis* and *P. zhennan*, volcano plots were generated for seeds and leaves (Figure 3A,B). In the seeds (ZJN1_vs_ZN1), a total of 1595 metabolites were detected, among which 525 were significantly upregulated, 577 were significantly downregulated, and 493 showed no significant change (Figure 3A and Supplementary Table S3). In the leaves (ZJN2_vs_ZN2), a total of 1640 metabolites were detected, with 79 metabolites upregulated, 1297 downregulated, and 264 non-significantly altered (Figure 3B and Supplementary Table S4). These results indicate a more pronounced downregulation of volatile metabolites in the leaves compared to the seeds. Hierarchical clustering heatmaps were constructed for the significantly differential metabolites (Figure 3C,D). Metabolites were organized according to their primary chemical classes, revealing distinct clustering patterns between *P. chekiangensis* and *P. zhennan* in both seeds and leaves. Terpenoids, aldehydes, and alcohols were major contributors to the separation between groups, suggesting that specific classes of metabolites may underlie the species-related aroma characteristics.

### 3.4. Functional Pathway Enrichment Analysis of Differential Volatile Metabolites

**Figure 4:**
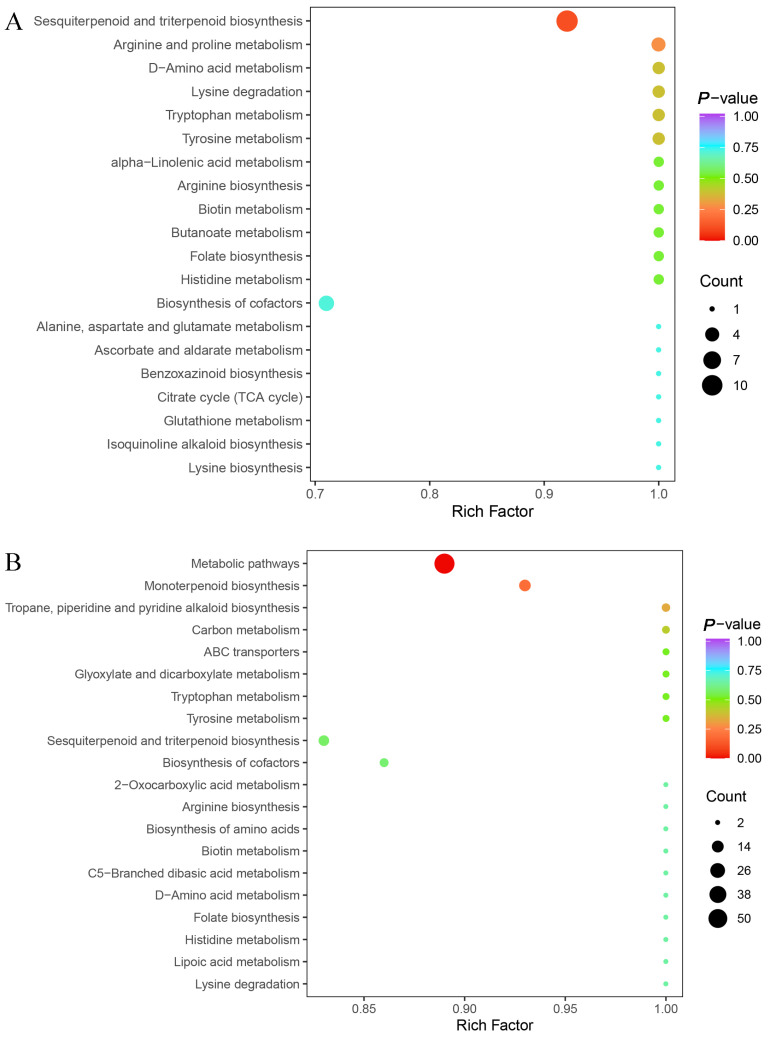
KEGG pathway enrichment analysis of differential volatile metabolites. (**A**) KEGG pathway enrichment of differential volatile metabolites between ZJN1 and ZN1, showing the top 20 significantly enriched pathways. (**B**) KEGG pathway enrichment of differential volatile metabolites between ZJN2 and ZN2, showing the top 20 significantly enriched pathways. The x-axis represents the rich factor, the size of the dots indicates the number of enriched metabolites, and the color represents the significance level (*p*-value).

To investigate the biological functions associated with differential volatile metabolites, KEGG pathway enrichment analyses were performed separately for seeds and leaves (Figure 4). In seeds (ZJN1_vs_ZN1), the top 20 enriched pathways included “Biosynthesis of secondary metabolites,” “Metabolic pathways,” “Monoterpenoid biosynthesis,” “Sesquiterpenoid and triterpenoid biosynthesis,” “Alpha-linolenic acid metabolism,” and “Phenylpropanoid biosynthesis” (Figure 4A). These enriched terms suggest that metabolites differentiating the two species are mainly involved in secondary metabolic processes. In leaves (ZJN2_vs_ZN2), differential metabolites were significantly enriched in pathways such as “Terpenoid backbone biosynthesis,” “Monoterpenoid biosynthesis,” “Sesquiterpenoid and triterpenoid biosynthesis,” “Flavonoid biosynthesis,” and “Phenylpropanoid biosynthesis” (Figure 4B). The greater representation of terpenoid- and phenylpropanoid-related metabolites in leaves is consistent with their distinct aroma profiles and may reflect tissue-related metabolic allocation. Overall, the KEGG enrichment results indicated that volatile metabolite differences between *P. chekiangensis* and *P. zhennan* are primarily associated with secondary metabolite biosynthesis, lipid metabolism, and aromatic compound production.

### 3.5. Sensory Flavoromics Analysis of Differential Volatile Metabolites

**Figure 5:**
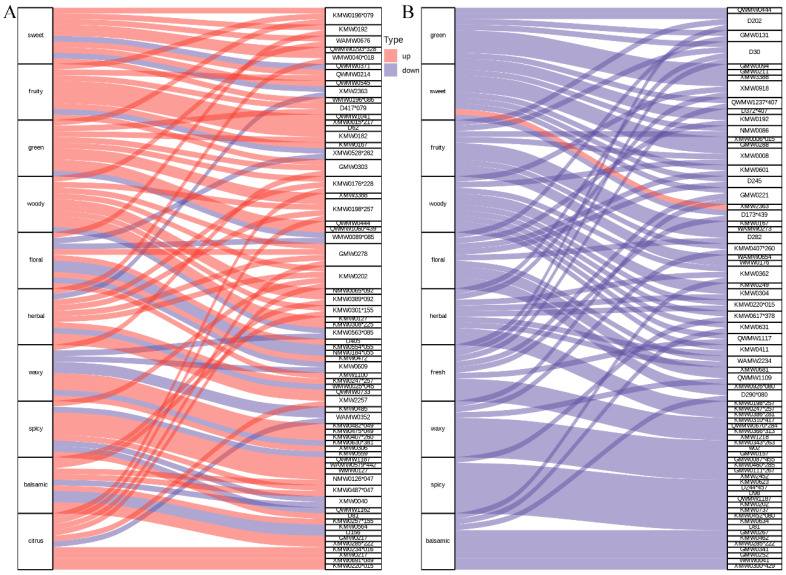
Sensory flavoromics analysis of differential volatile metabolites in seeds and leaves. (**A**) Sankey diagram displaying the top 10 sensory flavor descriptors associated with differential volatile metabolites between seeds of ZJN1 and ZN1. (**B**) Sankey diagram displaying the top 10 sensory flavor descriptors associated with differential volatile metabolites between leaves of ZJN2 and ZN2. In each diagram, the left side represents sensory attributes, and the right side represents associated volatile metabolites. A complete list decoding these abbreviations to show full compound names is provided in Supplementary Table S2. The width of the connecting lines reflects the number of metabolites annotated to each sensory descriptor.

To interpret the aroma implications of the differential volatile profiles between *P. chekiangensis* and *P. zhennan*, a flavoromics analysis was conducted based on annotated sensory descriptors of volatile metabolites (Figure 5 and Supplementary Table S2). In the seeds (Figure 5A), the top 10 sensory descriptors associated with differential metabolites included “sweet,” “fruity,” “green,” “woody,” “floral,” “herbal,” “waxy,” “spicy,” “balsamic,” and “citrus.” Among them, floral and fruity notes were linked to the highest number of metabolites, suggesting that seeds are predominantly characterized by sweet and pleasant aromatic attributes. In the leaves (Figure 5B), the dominant sensory descriptors were “green,” “sweet,” “fruity,” “woody,” “floral,” “herbal,” “fresh,” “waxy,” “spicy,” and “balsamic.” Compared with seeds, leaf volatiles exhibited a relatively stronger association with green, herbal, and fresh notes, indicating a greater accumulation of metabolites contributing to leafy and natural aromas. Overall, the aroma flavoromics analysis revealed distinct aroma profiles between tissues, with seeds exhibiting more sweet and fruity characteristics, while leaves displayed enhanced green, herbal, and fresh aromatic features. These distinct aroma signatures further support the organ- and species-related differentiation of aroma profiles in *Phoebe* species.

### 3.6. Venn Diagram Analysis Reveals Species-Related and Organ-Related Core Volatile Metabolites

**Figure 6:**
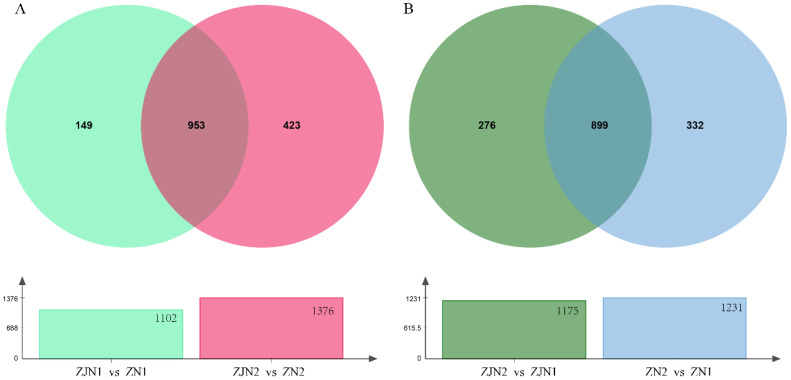
Venn diagram analysis of differential volatile metabolites highlighting species-related and organ-related differences. (**A**) Venn diagram comparing differential volatile metabolites between seeds (ZJN1_vs_ZN1) and leaves (ZJN2_vs_ZN2) of *P. chekiangensis* and *P. zhennan*. The overlapping metabolites represent species-related core volatile differences conserved across tissues. (**B**) Venn diagram comparing differential volatile metabolites between seeds and leaves of *P. chekiangensis* (ZJN2_vs_ZJN1) and *P. zhennan* (ZN2_vs_ZN1).

To identify conserved volatile metabolites associated with species and organ differences, Venn diagram analyses were conducted across the key comparison groups (Figure 6 and Supplementary Table S5). In the species comparison (ZJN1_vs_ZN1 vs ZJN2_vs_ZN2), a total of 953 differential metabolites were shared between seeds and leaves (Figure 6A), representing core species-related volatile markers. Additionally, 149 metabolites were unique to the seed comparison (ZJN1_vs_ZN1), and 423 metabolites were unique to the leaf comparison (ZJN2_vs_ZN2), suggesting that tissues also contribute to species-related volatile differentiation. In the organ comparison (ZN2_vs_ZN1 vs ZJN2_vs_ZJN1), 899 metabolites were consistently different between seeds and leaves across both species (Figure 6B), indicating conserved organ-related volatile differences. Meanwhile, 332 metabolites were unique to *P. zhennan* (ZN2_vs_ZN1), and 276 metabolites were unique to *P. chekiangensis* (ZJN2_vs_ZJN1), highlighting the species-related aspects of their organ-based volatile profiles. Overall, the Venn diagram analysis revealed both shared and unique volatile metabolites involved in species divergence and organ specialization, providing a basis for understanding the metabolic regulation of aroma characteristics in *Phoebe* species.

## 4. Discussion

Volatile metabolites are key mediators in plant–environment interactions, contributing to chemical defense, ecological signaling, and the sensory qualities of plant-derived products [3,4,5]. In the present study, we employed untargeted GC-MS-based metabolomics integrated with rOAV screening and sensory annotation to investigate the volatile profiles of two *Phoebe* species of ecological and ornamental importance, namely *P. zhennan* and *P. chekiangensis*. Our findings revealed extensive volatile diversity, with pronounced organ- and species-related patterns that reflect both functional differentiation and evolutionary divergence. A total of 1666 volatile metabolites were detected across all seed and leaf samples. These metabolites spanned multiple structural classes, with terpenoids, aldehydes, alcohols, ketones, and aromatic compounds dominating the profiles (Figure 1C). Such diversity is characteristic of Lauraceae and comparable to other aromatic trees such as *Cinnamomum* and *Litsea* [24,25]. The PCA and hierarchical clustering (Figure 1B, D) showed a clear separation of samples by both species and tissue type, with distinct clustering patterns observed for both *P. zhennan* and *P. chekiangensis*. Leaves generally exhibited a greater representation of terpenoid- and GLV-related compounds, often associated with herbivore resistance, allelopathy, or stress responsiveness [26,27]. In contrast, seeds were richer in volatiles with fruity, sweet, and floral descriptors, likely linked to seed protection and attraction of dispersal agents. The rOAV analysis identified 540 aroma-active metabolites, with a few standout compounds, such as two metabolites (3-Cyclohexene-1-methanethiol,α,α,4-trimethyl- and β-damascone) that displayed extremely high values in *P. zhennan* leaves (Figure 2). Their accumulation in *Phoebe* leaves may indicate active carotenoid turnover and oxidative pathways, potentially linked to light-induced signaling or stress responses. Sulfur-containing compounds, on the other hand, often contribute to pungent green or garlic-like aromas and are known to play antimicrobial or herbivore-deterring roles [28]. These findings underscore the importance of combining concentration data with odor threshold values to identify functionally relevant volatiles that dominate the perceived aroma profile.

The comparison of differential metabolite enrichment between species revealed additional layers of divergence. The Volcano plot analysis (Figure 3) showed that *P. zhennan* had a greater abundance of metabolites in seeds, while *P. chekiangensis* exhibited more downregulation in leaves, suggesting tissue-related repression or metabolic channeling. The KEGG enrichment analysis (Figure 4) supported this trend: *P. chekiangensis* seeds were enriched in monoterpenoid and phenylpropanoid biosynthesis pathways, while *P. zhennan* leaves showed greater involvement of sesquiterpenoid, triterpenoid, and terpenoid backbone biosynthesis. These observations are consistent with prior studies highlighting lineage-specific expansion and expression divergence of terpene synthase (TPS), cytochrome P450s, and BAHD acyltransferases in Lauraceae [29,30]. Species-dependent specialization of volatile pathways may reflect differing ecological niches, historical uses, or adaptive strategies. From a sensory perspective, our flavoromics analysis provided a functional interpretation of the metabolomic data. Sankey diagram visualization (Figure 5) revealed that *Phoebe* seeds were associated with “floral,” “fruity,” and “sweet” descriptors, whereas leaves displayed dominant “green,” “herbal,” and “spicy” attributes. These trends mirror structural class differences, as ionones, esters, and certain aldehydes commonly contribute to floral/fruity notes, while GLVs (green leaf volatiles) and terpenes drive green/herbal aromas [21,26,31]. Such divergence in volatile perception likely reflects underlying ecological roles, in which seed volatiles may attract mutualists, while leaf volatiles function primarily in defense.

The Venn diagram analysis revealed both conserved and tissue-related volatile metabolites across species. In the species comparison (ZJN1_vs_ZN1 and ZJN2_vs_ZN2), 953 shared metabolites were identified in both seeds and leaves (Figure 6A), likely representing core species-related volatiles driven by genetic regulation. These included mainly terpenoids and norisoprenoids, consistent with patterns reported in other Lauraceae plants [32,33]. An organ-level comparison (ZN2_vs_ZN1 and ZJN2_vs_ZJN1) identified 899 shared metabolites between species (Figure 6B), indicating conserved tissue-related profiles. Leaf volatiles tended to include GLVs and sesquiterpenes, while seeds were enriched in fruity and floral compounds, reflecting ecological roles such as defense or dispersal [26,27]. Meanwhile, species- and organ-related subsets highlight the joint influence of tissue function and evolutionary background. These findings provide a basis for chemotaxonomic markers, fragrance-oriented selection, and future gene–metabolite association studies.

In addition to species- and tissue-related comparisons, our findings can be contextualized with previous studies on Lauraceae plants and other aromatic trees. For example, *Litsea cubeba* fruits were reported to be enriched in monoterpenes like citral and limonene, whereas *Cinnamomum camphora* leaves and bark primarily accumulated linalool and camphor-type sesquiterpenes [34,35]. In contrast, *Phoebe* seeds in our study were dominated by norisoprenoids and aldehydes contributing floral and fruity aromas, while the leaves featured GLVs and diverse terpenoids linked to green and herbal attributes. These differences may reflect organ- related ecological roles such as seed dispersal or herbivore defense, as well as species-related adaptations to microenvironmental conditions or evolutionary lineage. Mechanistically, such divergence may result from the differential expression of terpene synthase (TPS), cytochrome P450, and acyltransferase genes that modulate downstream volatile diversity in a tissue-preferential manner, as suggested in Lauraceae transcriptome-based studies [29,30]. However, direct evidence linking gene expression to volatile output in *Phoebe* is still lacking and requires integrated transcriptomic and enzyme-level validation. As a limitation, our analysis relied solely on semi-quantitative GC-MS metabolomics of volatile extracts from two organs and two species under a single environmental condition. Without dry-weight normalization or water content correction, subtle changes in concentration may reflect physiological variation. In addition, although KEGG pathway enrichment provided useful biological context, it was based only on metabolite identity and relative abundance. Therefore, claims of pathway upregulation should be interpreted with caution. Beyond their ecological and biological relevance, the distinct volatile profiles observed in *Phoebe* seeds and leaves offer practical implications for the fragrance, essential oil, and wood product industries. For example, the enrichment of fruity and floral norisoprenoids in seeds suggests their potential as sources of natural aromatic additives or fragrance extracts. Meanwhile, the accumulation of green and herbal GLVs and terpenoids in leaves aligns with odor profiles desired in air fresheners or insect-repellent formulations. Tissue-related volatile markers may also aid in the chemotaxonomic authentication of Phoebe-derived products. Furthermore, these insights can support aroma-oriented breeding strategies or metabolic engineering to enhance desirable scent traits in wood materials or ornamental cultivars of Lauraceae species.

Overall, our findings demonstrate that the volatile profiles of *P. zhennan* and *P. chekiangensis* are shaped by a complex interplay between tissue identity, species background, and ecological function. By combining GC-MS metabolomics, rOAV quantification, KEGG pathway enrichment, and flavoromics interpretation, we provide an in-depth framework for dissecting aroma-related chemical diversity in woody plants. These insights have important implications for the conservation, commercial utilization, and metabolic engineering of aromatic tree species. It is worth noting that not all volatile compounds could be annotated in the KEGG database, particularly some sesquiterpenes and norisoprenoids. These unmapped compounds were excluded from the enrichment analysis, which may have limited the pathway coverage. Nevertheless, the annotated subset provided meaningful insights into the major metabolic routes underlying tissue- and species-related volatile differences. However, it should be noted that the KEGG enrichment analysis in this study was based solely on the presence and relative abundance of annotated metabolites. Therefore, the functional interpretations drawn from pathway enrichment should be viewed as suggestive of possible metabolic trends, rather than conclusive evidence of pathway activation or gene regulation, such as enhanced terpenoid biosynthesis. Future transcriptomic or flux-based analyses are needed to validate these metabolic inferences. Despite the in-depth analysis, this study is limited to two species and two organs under a single environmental condition. Further multi-seasonal, multi-site, and transcriptome-integrated investigations will be valuable for confirming the generality and underlying regulation of the volatile patterns observed here. Compared with previous studies of volatile metabolomes in aromatic trees across different regions, such as *Cinnamomum camphora* in Zhejiang [34] and *Litsea cubeba* in nine provinces of China [35] and other regions of the world, our study revealed a higher diversity of tissue-related volatile metabolites, particularly in non-wood organs like seeds and leaves. Many earlier reports focused mainly on essential oil extracts from mature wood or bark tissues and often targeted only a limited number of compounds. In contrast, our untargeted GC-MS approach identified over 1666 volatile compounds across tissues, enabling detailed chemotaxonomic and functional comparisons. Furthermore, the integration of rOAV analysis and sensory annotation provides a deeper interpretation of aroma-active profiles, which is often absent in earlier regional reports. Therefore, this study provides an in-depth advancement in the volatile metabolomic characterization of *Phoebe* species under subtropical East Asian environments, with methodological innovations that may be broadly applicable to other Lauraceae taxa and ecological contexts.

## 5. Conclusions

This study presents a systematic characterization of volatile metabolites in the seeds and leaves of *P. zhennan* and *P. chekiangensis*, using untargeted GC-MS-based metabolomics integrated with rOAV screening and sensory annotation. A total of 1666 volatile features were tentatively identified through spectral library matching, among which 540 were inferred to be aroma-active based on rOAV values calculated from literature-reported odor thresholds. Clear tissue- and species-related aroma profiles were revealed: seeds were enriched in floral and fruity compounds, while leaves featured more green and herbal volatiles. The KEGG enrichment and Venn diagram analyses suggested possible metabolic divergence and indicated putative diagnostic markers underlying this diversity. These findings improve our understanding of volatile allocation in different plant tissues and their potential ecological roles, such as in defense and in attracting pollinators. Furthermore, the results provide a valuable foundation for applications in fragrance product development, chemotaxonomic identification, and the future breeding or metabolic engineering of aromatic trees. Although this study provides new insights into the volatile diversity of *Phoebe* species, its reproducibility may be constrained by certain limitations, including the absence of absolute quantification, gene expression validation, and broader environmental sampling. Future research should aim to integrate transcriptomic data, conduct multi-seasonal and multi-site sampling, and apply standardized methods to enhance its robustness. These efforts will help validate the ecological and functional relevance of tissue-related volatile variation and provide a stronger basis for aromatic trait improvement in *Phoebe* species.

## Data Availability

The original contributions presented in the study are included in the article; further inquiries can be directed to the corresponding authors.

## Supplementary Materials

The following supporting information can be downloaded at: https://www.mdpi.com/article/10.3390/metabo15080526/s1, Figure S1: Geographical location of the sampling site. Table S1: List of 1666 detected volatile metabolites and their annotation information. Table S2: List of 540 aroma-active volatile compounds in *Phoebe* species. Table S3: Differentially accumulated volatile metabolites in seeds between *P. chekiangensis* and *P. zhennan* (ZJN1_vs_ZN1). Table S4: Differentially accumulated volatile metabolites in leaves between *P. chekiangensis* and *P. zhennan* (ZJN2_vs_ZN2). Table S5: Lists of differentially accumulated volatile metabolites based on pairwise Venn diagram comparisons.

## Abbreviations

The following abbreviations are used in this manuscript:

VOCs	Volatile Organic Compounds
GC-MS	Gas Chromatography–Mass Spectrometry
KEGG	Kyoto Encyclopedia of Genes and Genomes
PCA	Principal Component Analysis
HCA	Hierarchical Cluster Analysis
QC	Quality Control
SIM	Selected Ion Monitoring
SPME	Solid-Phase Microextraction
TPS	Terpene Synthase
GLVs	Green Leaf Volatiles
ZN	*Phoebe zhennan*
ZJN	*Phoebe chekiangensis*
