# Supplementary material for: Dissecting Organ-Specific Aroma-Active Volatile Profiles in Two Endemic Phoebe Species by Integrated GC-MS Metabolomics
Source: Metabolites. 2025 Aug 3;15(8):526. doi: 10.3390/metabo15080526 (PMC12388051; doi:10.3390/metabo15080526)
Supplement: Supplementary file 1 [file Supplementary Figure S1.pdf › Supplementary Figure S1.pdf]

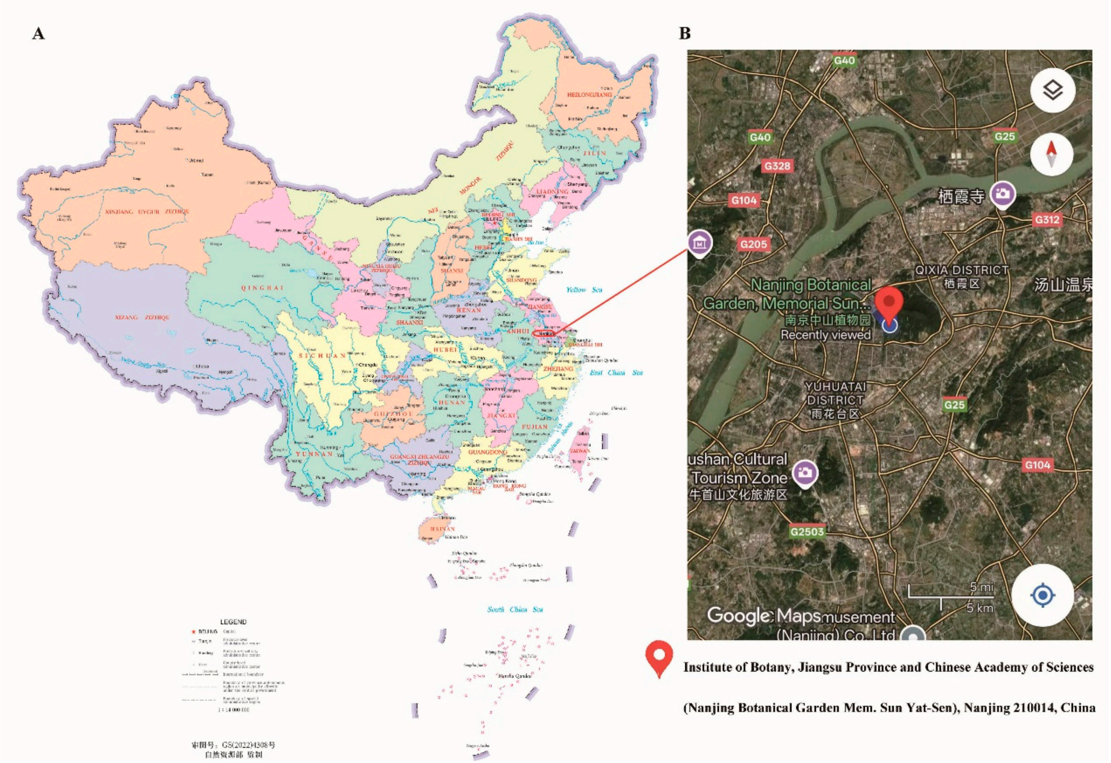

Geographical location of the sampling site.

(A) Map of China showing the location of Nanjing in the eastern region of the country. (B) Enlarged Google map of Nanjing highlighting the sampling site at Institute of Botany, Jiangsu Province and Chinese Academy of Sciences (Nanjing Botanical Garden Mem. Sun Yat-Sen), where mature seeds and leaves of *Phoebe zhennan* and *Phoebe chekiangensis* were collected.
